# Insight into Risk Factors, Pharmacogenetics/Genomics, and Management of Adverse Drug Reactions in Elderly: A Narrative Review

**DOI:** 10.3390/ph16111542

**Published:** 2023-11-01

**Authors:** Carlo Maria Bellanca, Egle Augello, Anna Flavia Cantone, Rosaria Di Mauro, Giuseppe Antonino Attaguile, Vincenza Di Giovanni, Guido Attilio Condorelli, Giulia Di Benedetto, Giuseppina Cantarella, Renato Bernardini

**Affiliations:** 1 Department of Biomedical and Biotechnological Sciences, Section of Pharmacology, University of Catania, 95123 Catania, Italy; uni318437@studium.unict.it (C.M.B.); uni365053@studium.unict.it (E.A.); anna.cantone@phd.unict.it (A.F.C.); peppettg@hotmail.it (G.A.A.); condorelliguido@icloud.com (G.A.C.); gcantare@unict.it (G.C.); bernardi@unict.it (R.B.); 2 Clinical Toxicology Unit, University Hospital of Catania, 95123 Catania, Italy; 3 Dipartimento del Farmaco, ASP Trapani, 91100 Trapani, Italy; rosariadimauro81@gmail.com (R.D.M.); vinnucciadgv@gmail.com (V.D.G.)

**Keywords:** adverse drug reactions, older adults, risk factors, drug interactions, prescription appropriateness

## Abstract

The European Medicine Agency (EMA) has defined Adverse Drug Reactions (ADRs) as “a noxious and unintended response to a medicine”, not including poisoning, accidental, or intentional overdoses. The ADR occurrence differs based on the approach adopted for defining and detecting them, the characteristics of the population under study, and the research setting. ADRs have a significant impact on morbidity and mortality, particularly among older adults, and represent a financial burden for health services. Between 30% and 60% of ADRs might be predictable and preventable, emerging as a result of inappropriate prescription, drug chemistry inherent toxicity, cell-specific drug toxicity, age- and sex-related anomalies in drug absorption, distribution, metabolism, and elimination (ADME), and drug–drug interactions (DDIs) in combination therapies or when a patient is treated with different drugs for concomitant disorders. This is particularly important in chronic diseases which require long-term treatments. Rapid developments in pharmacogenetics/genomics have improved the understanding of ADRs accompanied by more accurate prescriptions and reduction in unnecessary costs. To alleviate the burden of ADRs, especially in the elderly, interventions focused on pharmaceutical principles, such as medication review and reconciliation, should be integrated into a broader assessment of patients’ characteristics, needs, and health priorities. Digital health interventions could offer valuable solutions to assist healthcare professionals in identifying inappropriate prescriptions and promoting patient adherence to pharmacotherapies.

## 1. Introduction

The burden of individuals affected by multiple chronic conditions, referred to as multimorbidity [1], is increasing proportionally to the lengthening of life expectancy of the overall population. Treating these different comorbidities often requires the use of multiple medications. Consequently, it is common for older individuals to be exposed to various drugs, a condition defined as polypharmacy. Notwithstanding, unanimous consensus on the definition of polypharmacy has not yet been established, as this term is commonly used by researchers to refer to the prescription of a minimum of five to ten medicaments [2]. In Europe, the estimated global prevalence of polypharmacy was 32.1%, defined as the administration of five or more medicinal products [3]. However, the prevalence of polypharmacy varies depending on its definition as well as the assessment method used, but also on the country, setting, and age subgroup. In Italy, 49% of patients older than 65 years were found to receive polypharmacy (a minimum of 5 concurrent medicinal products) and 11.3% had excessive polypharmacy (a minimum of 10 drugs), with greater prevalence in the south of Italy [4,5,6].

The growth of this phenomenon in developed countries can be attributed not just to the aging population but also to the availability of new medicinal products for chronic diseases. Additionally, Pharma companies and pharmaceutical commercial agents can influence the prescription behaviour of medical doctors. A recent systematic review highlighted that interactions with pharmaceutical industry representatives may influence physician prescribing habits and contribute to irrational prescription practices [7]. The concept of “pharmaceuticalization” has been adopted to underline the significance of pharmaceutical manufacturing in contemporary society [8]. The latter also explains the growing trend of purchasing and taking medicinal products out of the physician’s advice, such as over-the-counter medications, herbal remedies, dietary supplements, or drugs bought online with no prescription needed [9]. Despite variations in estimates of polypharmacy and reasons accounting for its rise, reports are consistently linked to an elevated potential for drug–drug or drug–disease interactions, adverse reactions, potentially inappropriate medications (PIMs), geriatric syndromes, falls, and death [10,11].

Adverse drug reactions (ADRs), which are prevalent among geriatric patients, can emerge as a result of inappropriate prescription, drug chemistry inherent toxicity, cell-specific drug toxicity, age- and sex-related anomalies in drug absorption, distribution, metabolism, and elimination (ADME), and drug–drug interactions (DDIs) in combination therapies or when a patient is treated with different drugs for concomitant disorders [12]. This is particularly important in chronic diseases which require long-term treatments especially, as discussed above, in the elderly [13,14].

Rapid developments in pharmacogenomics have improved the understanding of ADRs accompanied by more accurate prescription and reduction in unnecessary costs [15]. Both drug resistance and drug toxicity are closely associated with potentially identifiable dysfunctions in pharmacoepigenetic apparatus [16]. Since cardiovascular diseases, cancer, and central nervous system (CNS) disorders account for over 80% of morbidity and mortality in developed countries, with 15–20% of direct costs related to pharmacological treatment (<30–40% of cost-effectiveness; >50% of ADRs), it seems reasonable to assume that the incorporation of pharmacogenomic testing prior to treatment would be of great benefit in terms of costs, quality of life (QoL), and optimization of therapeutic resources [17,18,19].

Observance to prescribing recommendations, appropriate monitoring, and regular medication review and reconciliation can decrease the number of improper prescriptions [20].

Therefore, reducing the number of prescribed remedies for elderly adults may improve their health, QoL, reduce hospitalizations, and decrease mortality rates. Within this framework, strategies, practices, and resources to minimise iatrogenic harms in multimorbid elderly patients, by reducing their medication burden, are strongly recommended [21,22,23].

## 2. Objectives

ADRs are a critical health issue, most importantly in the elderly, who are often suffering from a number of medical conditions that require a polypharmacy treatment plan. This narrative review aims to provide an overview of the main risk factors associated with the occurrence of ADRs and navigate through the possible strategies currently available to reduce their incidence, including the compelling implementation of pharmacogenetic/genomic procedures. Indeed, adherence to physician recommendations, proper surveillance, and periodic review and reconciliation of medications may limit the number of inappropriate prescriptions, thereby promoting better health and QoL, fewer hospital admissions, and lower mortality rates.

## 3. Methods

A search of the relevant literature (up to July 2023) was conducted on MEDLINE (PubMed) and Google Scholar by applying the medical subject headings (MeSH) terms “elderly”, “prescription appropriateness”, “drug–drug interactions”, “drug–gene interactions”, “drug–drug–gene interactions”, “adverse drug reactions”, “multimorbidity”, “pharmacokinetics/dynamics”, ”pharmacogenetics/genomics”, “medication review”, “medication reconciliation”, “pharmacoepidemiology”, and “digital tools”. From the web-based search, we selected peer-reviewed, full-text, and English language manuscripts. Meta-analysis, systematic review, observational studies, and reviews were included. We excluded single case studies, paediatric studies, and non-peer reviewed publications. Each selected paper was preliminarily examined by both senior authors (via abstract reading), downloaded, and summarised.

## 4. Adverse Drug Reactions

### 4.1. Definition and Classification

The European Medicine Agency (EMA) has defined Adverse Drug Reactions (ADRs) as “a noxious and unintended response to a medicine”. Consequently, they can be described as “any harmful, undesired, or unintended response to a therapeutic agent, which may be expected or unexpected and may occur at dosages used for prophylaxis, diagnosis, or therapy of disease, or for modifying physiological functions”. On the other hand, ADRs do not include poisoning, accidental, or intentional overdoses [24].

There are several classification systems for ADRs. The first, suggested by Thomson and Rawlins in 1981, distinguishes ADR into Type A and Type B. Type A reactions happen in response to drugs administrated at therapeutic doses being the result of an abnormal response of an otherwise normal pharmacological effect. They are frequent, but it is unlikely that they lead to a fatal event. Type B reactions are not related to drug’s pharmacodynamics or dosage and are often lethal. The integration of four additional types of reaction has further updated this classification: Type C, related to the cumulative dose of a prolonged pharmacological treatment; Type D, as a consequence to the timing of a treatment; Type E, associated with the withdrawal of a given drug; and Type F, which occurs if therapy appears futile [25,26,27].

The Dose, Time and Susceptibility (DoTS) and EIDOS schemes provide alternative classification and are mutually complementary. The former considers drug dose, time since onset of the reaction, and if inherent susceptibility factors have been implicated in the response. In addition, a description of reactions’ clinical aspects is provided. The latter classification becomes a useful tool in pharmacovigilance and for recognizing new adverse reactions [28]. The EIDOS mechanistic classification considers Extrinsic chemical species (E) supposed to initiate the effect; Intrinsic chemical species (I) involved; Distribution (D) of these species in the body; Outcome (O); and Sequelae (S), which represent the final adverse drug reactions [29].

Given the wide range of manifestations, ADRs may be wrongly interpreted as either signs or symptoms of a pathological condition, rather than as effects of pharmacotreatments. An ADR can manifest itself as syncope, falling, or bleeding from the gastrointestinal tract [30]. In evaluating a patient’s medication history, particularly in older individuals, healthcare professionals should be careful to recognise a possible association between a clinical manifestation and a specific medication. An ADR Probability Scale, developed by *Naranjo* et al., may be useful in assessing and classifying the causal relationship between the ADR and the suspected drug. The ten-item scale can be easily compiled in a clinical setting. The overall score indicates the probability that an adverse effect is related to a drug reaction [31]. The classification methods described above are summarised in Table 1.

### 4.2. Epidemiology

The occurrence of ADRs differs based upon the approach adopted for their definition and detection, the characteristics of the population under study, and research settings. Most of the existing studies are primarily focused on hospital environment, as they provide an opportunity to closely monitor hospitalized patients for the presence or the development of ADRs. Furthermore, these patients typically exhibit frailty and susceptibility to acute diseases, which can lead to an increased number of medications prescribed, as well as a heightened vulnerability to adverse effects, thereby amplifying the severity of drug-related illnesses [32].

According to European Commission estimates, around 5% of all hospital admissions can be attributed to ADRs, and during their hospital stay; approximately 5% of hospitalized patients will experience an ADR. In Europe, in 2008, it was reported that 197,000 deaths were linked to ADRs [33].

More recently, a meta-analysis assessed that ADRs account for 8.7% (95% CI = 7.6–9.8%) of hospital admissions. Nonsteroidal anti-inflammatory drugs (NSAIDs) were among the most frequently drug classes associated with hospitalisation, with studies reporting rates between 2.5% and 33.3%. Beta-blockers (1.8–66.7%), antibiotics (1.1–22.2%), oral anticoagulants (3.3 to 55.6%), digoxin (1.6–18.8%), angiotensin-converting enzyme inhibitors (5.5–23.4%), oral antidiabetics (4.5–22.2%), and opioids (1.5–18.8%) were also associated with ADRs. Risk factors for hospital admission due to ADRs included number of medicines (in every study that investigated this variable), concomitant diseases, female gender, age, and inappropriate pharmacotherapy [34,35].

A recent exploratory review analysed 32 observational studies, both prospective and retrospective, from various settings in twelve different countries. The incidence of ADRs was assessed by measuring the number of hospital admissions due to ADRs, the total of ADRs during hospitalisation, and the number of ADRs in outpatient settings over a given interval. The results of the studies indicated an overall ADR rate of 3.6% at the time of hospital admission and 10.1% during hospitalisation. Five studies evaluated the incidence of ADRs in older people residing in the community and reported a wide range of estimates. The overall fatal ADR rate was approximately 0.5%, with type A reactions being the most commonly observed [36].

The frequency of ADRs in the long-term care sector has not been extensively studied. One prospective cohort study conducted in the USA examined long-term care residents and revealed that at least 14% of them experienced an ADR over a period of 12 months [37]. Another study explored ADR-related hospitalizations among nursing home residents and found that 15.7% of the 332 participants had undergone at least one hospitalization directly linked to the number of pharmacological treatments taken per day and the medications most frequently associated with these events were NSAIDs, psychotropic drugs, digoxin, and insulin [38]. Similarly, in a study involving American nursing home residents, the use of antipsychotics, anticoagulants, diuretics, and anti-epileptics was found to increase the risk of preventable adverse reactions, the most common of them have been delirium, over-sedation, and falls [39,40].

For the purpose of this review, it is important to highlight that twice as many patients aged 65 and over are hospitalised for clinical consequences of adverse drug reactions as their younger counterparts [41]. Moreover, *Dilles* et al. have shown that 60% of nursing home residence have still experienced ADRs, reinforcing them as a significant health concern in vulnerable elderly [42].

Ultimately, it is possible that the occurrence of ADRs in older adults is underestimated due to a substantial rate of under-reporting and the potential dismissal of new signs or symptoms as ADRs [43].

## 5. Adverse Drug Reactions in the Elderly Population: Risk Factors

The International Conference of Harmonization (ICH) considers individuals aged 65 years or older as a “special population” because the differences with regard to comorbidity, polypharmacy, pharmacokinetics, and greater susceptibility to ADRs than the younger adults [44].

ADRs have a significant impact on morbidity and mortality, particularly among the elderly population, and are a financial burden for healthcare systems. It is estimated that between 30% and 60% of ADRs might be predictable and preventable. Indeed, they often arise from medication errors such as improper indications, high dosages, or prolonged treatment duration, as well as non-compliance with prescribed regimens or inappropriate self-medication in elderly and vulnerable patients. In addition to higher incidence, ADRs in older adults are more likely to be severe and underreported. Furthermore, the mortality rate associated with ADRs is significantly greater in older patients compared to younger individuals [45,46].

The care pathways for elderly patients can significantly differ from those of younger individuals with the same medical condition, particularly when considering treatment options and choices. The primary objective is not just a matter of treating a pathological condition, it is instead maintaining their independence, social engagement, and overall quality of life to the greatest extent possible. As a result, physicians are advised to consider patients’ health trajectories and needs to establish realistic therapeutic goals [47,48]. Following diagnosis, the process of medical prescription is mostly driven by the necessity to prevent the clinical manifestations and complications of the disease, including its interactions with concurrent conditions and pharmacological treatments. For these reasons, physicians should tailor the clinical recommendations outlined in the guidelines for a patient’s specific characteristics, rather than adhering strictly to disease-specific protocols. Another critical aspect involves the frequent reassessment of the ongoing treatment’s appropriateness. As older individuals often experience unstable health courses, the benefit/risk ratio of individual therapies may fluctuate in response to changes in their clinical conditions [47].

This strategy becomes even more important when one considers that drugs developed so far are not designed for individual patients, but for the average population; therefore, while working for the huge population majority, they are ineffective or even toxic for a part of it [49].

Several studies indicate that older adults may exhibit a higher incidence of ADRs compared to younger ones, and advancing age itself can be considered as a risk factor for ADR occurrence. In light of these findings, *Stevenson* et al. have proposed the necessity of adopting a comprehensive approach to address ADRs among the elderly population, treating drug-related harm as a geriatric syndrome [50].

As previously stated, numerous factors associated with ripe old age can lead to an elevated risk of incurring an ADR, such as higher rates of polypharmacy, multimorbidity, reduced organ function, frailty, age-related pharmacokinetic and pharmacodynamic variations, and individual variability in pharmacogenomics/pharmacogenetics. In addition, over the past few years, there has been growing recognition of the significant impact of genetic variations in drug-metabolizing and drug-transporting proteins on the occurrence of ADRs among older individuals. This is in line with the prevailing understanding that genomic variants can contribute to ADRs and can be effectively utilized to predict an individual’s response to drugs, encompassing both effectiveness and potential toxicity [51,52].

### 5.1. Polypharmacy

A significant age-related factor contributing to the higher prevalence of ADRs in the older population is represented by polypharmacy; indeed, multiple medications are commonly prescribed to manage various health issues simultaneously. International estimates indicate that over 60% of elderly individuals are prescribed five or more medications at the same time. The potential harm from drug reactions and interactions rises with the greater number of medicinal products, and the total number of drugs taken per day becomes a significant risk factor for ADR-related hospitalizations [53,54,55]. For instance, the risk of experiencing ADRs is 13% for a person on two medications, increasing to 58% and 82% for those taking five or seven or more medications per day, respectively [14]. These factors partially explain as such the elevated risk of adverse reactions observed in the older population, underlining the need for cautiousness when prescribing new drugs. Specific guidelines have been developed in Italy by Onder and colleagues with the aim of providing recommendations, currently lacking, for the care management of patients most susceptible to polypharmacy and multimorbidity. Such indications may not only improve the quality of individual care, but also assist the clinician, health professionals, and caregivers. The emphasis is on the need for multidimensional assessment through personalised and interdisciplinary approaches to identify patients most vulnerable to negative outcomes associated with polypharmacy. A limitation may be that there is inadequate evidence that the number of drugs per se, rather than inappropriate prescribing, is the direct cause of adverse consequences [47].

The majority of ADRs in the elderly belong to Type A, which are predictable and preventable with adequate evaluation and monitoring. Thus, prudent prescribing practices are essential in reducing errors and minimizing the risk of ADRs considering patient susceptibilities, medication history, and exploring non-pharmacological or conservative options [14,56,57].

Notably, the eldest and frail individuals are frequently excluded from clinical trials, making it challenging to hypothesise the nature and incidence of adverse events. Moreover, guidelines predominantly focus on managing single diseases, so strict adherence to them when prescribing may be detrimental when dealing with older individuals with multiple comorbidities [58].

### 5.2. Multimorbidity

Multimorbidity refers to the coexistence of at least two chronic diseases in the same patient. Particularly in geriatrics, multimorbidity is a significant concern as it is closely linked to the occurrence of iatrogenic illnesses. Several studies have highlighted that the risk of ADRs escalates with an increasing number of chronic diseases. This can be attributed to various factors, including a higher likelihood of drug–disease interactions, as medications used for one condition may worsen symptoms of other underlying disorders [59,60,61].

### 5.3. Changes in Drug Metabolism

The aging process significantly affects the body’s homeostasis, in one with physiological processes, that, possibly, increase the risk of iatrogenic events [62], therefore pharmacokinetic alterations due to age, as well as factors like multimorbidity, frailty, and polypharmacy, as mentioned above, may play a critical role in this circumstance [57,63,64]. Changes in pharmacokinetics impact drug metabolism and clearance, thereby heightening the risk of ADRs, or altered drug responsiveness [65]. Modifications in the distribution volumes of drugs, attributable to a lower body fluid content and differences in lipid distribution, can contribute to the prolongation of the half-life of a given drug, thus escalating the probability of toxicity. In patients on polypharmacy, drug metabolism can also be affected by interactions with CYP450 enzymes. A cross-sectional study of eighty-year-old institutional and community residents revealed that 72.2% of individuals exhibited potential CYP drug-to-drug interactions that affected not only their functional performance and mobility, but also their self-perception of health [62,66]. So far, the impact of gender on the incidence of ADRs has been clearly demonstrated in a large number of studies. In particular, a systematic analysis aimed at assessing the extent of sex differences in ADRs across a wide range of treatments has found that slightly less than half of the medicines investigated (307 vs. 668) show a different sex-related rate of ADRs [67].

Gender-based differences in genetics, immunology, pharmacokinetics, and pharmacodynamic may account for the existing variability of ADRs, with women being generally more susceptible than men [68].

In addition, levels of sex steroid hormones, which contribute to differences in drug response, have been shown to change with age, thus potentially directly and indirectly affecting drugs’ ADME. Understanding physiological variations between the two sexes would enable personalised medicine to move forward [69,70].

### 5.4. Geriatric Syndromes

Geriatric syndromes encompass a range of conditions such as falls, delirium, cognitive impairment, orthostatic hypotension, incontinence, and chronic pain, which can reduce the potential benefits of pharmacological treatments [71,72], increase the risk of ADRs, and contribute to inappropriate prescriptions [73]. For instance, older adults taking oral antidiabetic medications face a heightened vulnerability to hypoglycaemia, thereby raising the risk of falls. Certain medications such as anticonvulsants, antidepressants, and some anti-Parkinsons drugs have been linked to an elevated risk of delirium and incontinence. Treatments for chronic pain, such as opioid agonists, have also been associated with delirium and an increased risk of falls. Moreover, some treatments may have indirect fatal consequences, as in patients with atrial fibrillation who are at high risk of falls, as anticoagulant therapy has been shown to elevate the risk of intracranial bleeding [74].

### 5.5. Pharmacogenetics/Genomics Variability

Pharmacogenetics/genomics is an evolving field with significant potential regarding its clinical application in tailoring therapy to optimize effectiveness and minimize the risk of adverse reactions. Pharmacogenetics focuses on the study of genetic factors that contribute to individual variations in drug response, while pharmacogenomics involves the genome-wide analysis of genetic determinants of drug efficacy and toxicity. The main distinction between pharmacogenetics and pharmacogenomics lies in the former’s emphasis on examining a few specific genes, whereas the latter encompasses the study of genes across all chromosomes [75,76,77]. The advancements in this field can be largely attributed to the increasing availability of information about the human genome, which has been made accessible to the entire scientific community since the completion of the human genome project in 2003. Thanks to progresses in bioinformatics, the massive volume of data derived from human genome sequencing has been organized, processed, and catalogued in databases [78].

A pharmacogenetic test must meet certain necessary criteria to be used in clinical practice. First, an established association between a genotype and the response to a specific drug is needed, either in the general population or a specific subgroup. The test must demonstrate both clinical and analytical validity. The method used to determine the genotype should exhibit sufficient sensitivity, specificity, and predictive values. Lastly, there should be scientific evidences supporting the utility of the test, meaning its application in a clinical setting should enhance treatment response, patient compliance, and ultimately improve the benefit/risk ratio associated with drug administration [79].

The majority of phase I reactions, such as oxidation, reduction, and hydrolysis, involved in drug metabolism to activate a prodrug or convert parent drugs to active or inactive metabolites, are facilitated by the cytochrome P450 superfamily of haemoproteins [80]. Among those, CYP1A2, CYP2C9, CYP2C19, CYP2D6, CYP2E1, CYP3A4, and CYP3A5 are considered the most significant for drug metabolism. They are responsible for approximately 90% of the systemic clearance and bioavailability of human drugs [81]. Medicinal products interacting with the CYP450 isoenzymes can be classified as substrates, inhibitors, or inducers. Inhibitors can be further characterized as being weak, moderate or potent [82].

Functional CYP polymorphisms encompass a wide range of genetic variations, including gene deletions and duplications, frame shift mutations, amino acid changes, intronic mutations (leading to altered splicing sites), and copy number variations in functional gene copies. Generally, the population can be classified into three major phenotypes based on a specific CYP450 enzyme: ultrarapid metabolizers, who possess more than two active genes encoding a particular P450 enzyme; extensive metabolizers, who carry two functional genes; poor metabolizers, lacking functional enzymes due to defective or deleted genes. Additionally, an intermediate metabolizer phenotype is often considered, encompassing individuals with either one functional and one defective allele, or two partially defective alleles [52,83,84].

Hence, variation in genes encoding drug-metabolising enzymes, drug transporters, and drug targets affects drug disposition and action, contributing to variability in drug response and in the development of ADRs. Thus, it may be useful in clinical practice to recognise the importance of genetic variation in contributing to the potential occurrence of ADRs, given the large number of genes involved in drug metabolism and transport. However, patients, especially elderly, may receive multiple inhibitors of a given cytochrome or an inhibitor and an inducer of the same one, making it difficult to predict the clinical relevance of these interactions. Furthermore, it is not enough to identify a clinically significant interaction; it would also be necessary to inform patients, modify the treatment plan, and perform regular follow-up to assess the safety and efficacy of the new pharmacotherapy. Nonetheless, in light of recent advances in pharmacogenetics/genomics, it would be desirable to revise the conventional understanding of DDIs encompassing the influence of genetic variations. Indeed, by applying pharmacogenetics/genomics a profile of a patient’s gene variations may be created, prior to administration of a drug. To our knowledge, to date, a single open label, multicentre, controlled, cluster-randomized, crossover implementation study investigating the beneficial impact of pharmacogenomics testing, has been performed. Despite its clinical utility not yet being established, encouraging results have emerged showing that clinically relevant ADRs reduced by about 30% with pharmacogenetics-guided prescribing [85].

**Figure 1:**
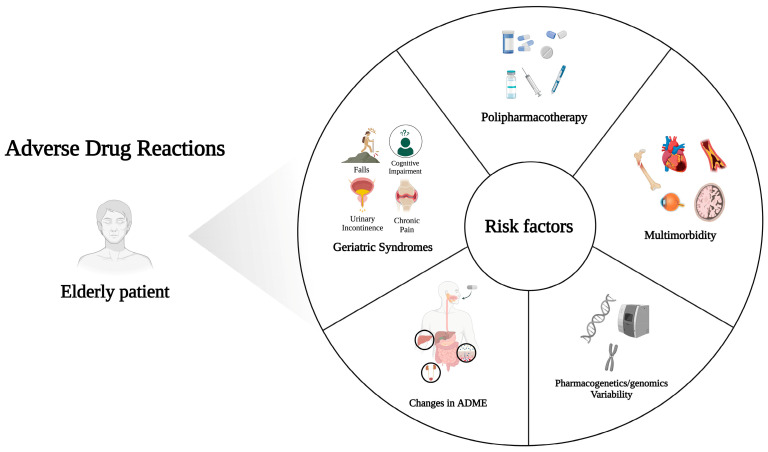
Summary of risk factors leading to ADRs in elderly patients. Created with BioRender.com; accessed on 19 October 2023.

A summary of risk factors involved in ADRs occurrence is provided in Figure 1.

#### 5.5.1. Drug–Drug Interactions (DDIs), Drug–Gene Interactions (DGIs), and Drug–Drug–Gene Interactions (DDGIs)

Drug–drug interactions (DDIs) can be divided into pharmacodynamic (PD) and pharmacokinetic (PK) interactions. PD interactions occur when drugs cause additive or antagonistic pharmacological effects that influence safety and/or efficacy. The co-administration of warfarin and NSAIDs is an example of a PD interaction, as their concomitant use may increase the risk of bleeding. PK interactions may be due to changes in absorption, distribution (protein and tissue binding), metabolism, and excretion [86].

In terms of PK, drug–gene interactions (DGIs), occur when an individual carrying one or more variant forms of a gene encoding a drug metabolizing enzyme or drug transporter with altered function receives a drug that is a substrate for the given enzyme or transporter [87].

Drug–drug–gene interactions (DDGIs) arise when both drugs and an individuals’ genetic profile alter the efficacy and/or safety of a specified medicinal product [88]; this notion will help to understand the phenoconversion interactions described below.

It is worth noting that in the real-world setting the interpretation of metabolic phenotypes should be evaluated considering DDGIs as a consequence of polypharmacy, especially in elderly. Yet the majority of pharmacogenetic recommendations are still based on the more well documented single gene–drug interaction, although the concomitant administration of another medication could influence the individual response [89].

Interaction mechanisms can be divided into three main categories: induction, inhibitory, and phenoconversion. The first two refer to any interactions that may impact PK of the victim drug, either by increasing or decreasing its concentrations. The latter can arise from the administration of a perpetrator drug that affects the metabolism or transport of the victim drug, as well as the presence of genetic variants leading to loss- or gain-of-function (LOF or GOF) in enzymes responsible for the metabolism or transport of the victim drug, or even a combination of both factors.

A phenoconversion can occur when the combined effect of the interacting drug and the genotype produces opposing effects, resulting in a temporary shift in phenotype [90].

As the management of DDIs largely depends on the clinical impact and severity of the interaction, many tools are available to determine their clinical significance. However, there is poor agreement among the current resources and a standardised classification method would be warranted. More specifically, the *British National Formulary* marks with bullet points potentially harmful drug pairs which should be prescribed cautiously, under appropriate monitoring, or avoided altogether [91]. The *Micromedex Drug–Reax System* categorises interactions into three degrees of severity, major, moderate, and minor, and the strength of the reporting into five categories—excellent, good, fair, poor, and unlikely [92]. The *Drugs.com Drug Interaction Checker* (DDIC) classifies interactions into four severity levels: major, moderate, minor, and unknown [93]. Vidal’s *Interactions médicamenteuses* comprises four seriousness grades according to the recommended clinical management—contraindicated, avoid, precaution, and “take into account” (i.e., no specific recommendation) [94]. *Drug Interaction Facts* rates interaction severity into three levels—major, moderate, and minor—and the degree of documentation into five—established, probable, suspected, possible, and unlikely—by combining these two categories. It also ranks each interaction from 1 to 5 in terms of importance [95].

##### Induction, Inhibitory, and Phenoconversion Interactions

Increased metabolism of active drugs due to the presence of an enzyme inducer or GOF variant can lead to a decrease in the effectiveness of the substrate drug. Prodrugs exhibit an opposite effect. When an enzyme-inducing drug or GOF variant are involved in their metabolism, elevated plasma levels of active metabolites may occur, leading to increased side effects and/or efficacy. For instance, individuals with CYP2C19*17 GOF variants experience enhanced conversion of clopidogrel to active metabolites, which in turn reduce the occurrence of cardiovascular events, but may increase the likelihood of bleeding episodes, which would be particularly risky in older and frail adults. Indeed, a five-year review of spontaneous pharmacovigilance reports elucidated an increased susceptibility to ADRs related to antithrombotic medicinal products [96]. Furthermore, *Dubrall* et al. emphasise the need to continuously monitor the prescription of antithrombotics in the elderly, as they account for a high proportion of ADRs in this cohort of patients [97]. If a CYP1A2, CYP2C9, and/or CYP3A4 inducer is co-administered, this is expected to enhance the efficacy of clopidogrel, but also an increase in bleeding risk [98,99,100,101,102].

The inhibitory effects of drugs and genotype can influence substrate metabolism by affecting either the same metabolizing enzyme or different metabolic pathways through drug and genotype interactions. Generally, individuals classified as poor metabolizers are expected to have the highest plasma concentration of substrate drug when co-administered with inhibitor, compared to other metabolic genotypes. For instance, the co-administration of simvastatin, a CYP2C9 inhibitor, with warfarin, a CYP2C9 substrate, has demonstrated a reduction in warfarin dosage requirements, particularly in CYP2C9*3 carriers, with a significantly higher percentage compared to noncarriers (29% vs. 5%, respectively) [103]. Nevertheless, the inhibitory effects of drugs and genotypes do not always exhibit additive behaviour. Genetically poor metabolizers are likely to experience only limited additional enzyme inhibition when an inhibitory drug is administered. For example, a statistically significant increase in rabeprazole, a CYP2C19 substrate, in plasma levels were observed in both normal metabolizers and carriers of the heterozygous genotype after treatment with fluvoxamine (CYP2C19 inhibitor). Nonetheless, no further clinically meaningful increase was detected in poor metabolizers, who already displayed highest rabeprazole plasma levels [104].

If a drug is metabolized by multiple CYP enzymes, inhibiting only one of those (via drug or genotype) may have minimal impact, due to the presence of alternative pathways. However, when both the genotype and the interacting drug affect different routes of metabolism, interactions may be significant.

A large proportion of prodrugs require specific CYP enzymes to become therapeutically active, taking clopidogrel as an example, which relies on the activation of CYP1A2, CYP2B6, CYP3A4, CYP2C9, and CYP2C19 [105]; individuals carrying LOF variants in one or more of these genes, and who are co-treated with inhibitors of these enzymes, face an increased risk of treatment resistance. For example, carriers of CYP2C19*2 and/or *3 alleles who receive clopidogrel alongside proton pump inhibitors, CYP2C19 inhibitors, are more prone to experience reduced efficacy of clopidogrel. Additionally, the introduction of a third risk factor, such as calcium channel blockers, CYP3A4 inhibitors, is associated with an even greater reduction in clopidogrel effectiveness [106,107].

A temporary change in phenotype can occur when the effect of a perpetrator drug is opposed to the genetic one. For instance, individuals with reduced function CYP2C9 variants exhibit decreased metabolism of tolbutamide, a CYP2C9 substrate. However, co-administration of rifampicin, an inducer of CYP2C9, in these patients counteracts the genetic effect, leading to a twofold increase in tolbutamide clearance [108]. On the other hand, when proton pump inhibitors are used alongside clopidogrel, a phenoconversion occurs, transforming individuals with genetically determined ultra-rapid metabolism into poor metabolizers, as evidenced by the loss of clopidogrel’s efficacy [109].

The bright side of phenoconversion interactions is that genetically determined phenotypes can be restored to normal by introducing medications with opposing effects on metabolism. As an example, resistance to nortriptyline, a CYP2D6 substrate, caused by excessively rapid metabolism, can be successfully reversed and normalized by adding paroxetine a CYP2D6 inhibitor, resulting in a restoration of therapeutic plasma levels of nortriptyline [110].

#### 5.5.2. Drug–Drug-Transporters–Genes Interactions (DDTGIs)

Drug transporters regulate the “movement” of pharmaceutical compounds to and from various body districts. Key locations where these transporters impact drug pharmacokinetics include liver, kidneys, blood–brain barrier (BBB), and intestine.

Transporters can be classified into two main categories, namely efflux transporters (divided into group I and group II accordingly to transport direction) and uptake transporters (group III).

Similar to the drug metabolizing enzyme scenario, these interactions may be intensified or reversed, via inhibitory/induction or phenoconversion pathways [111]. Nonetheless, some issues need to be considered in respect to drug–drug-transporters interactions in clinical practice. Indeed, there are a very limited number of drugs, if any, whose carrying depends on a single transporter; the victim drug may be a substrate of other uptake or efflux transporters. Furthermore, a large proportion of victim drugs are not only substrates of one or more transporters, but also metabolised by one or more phase I and/or phase II enzymes. Perpetrator drugs are also often not specific for a single transporter, but they may inhibit or induce other transporters and/or drug metabolising enzymes. Thus, it is still difficult to obtain clear data on in vivo contributions to overall changes in the pharmacokinetics of victim drugs for each specific transporter-mediated drug–drug interactions. Additionally, during drug development, not all theoretically possible drug combinations can be tested for transporter-mediated drug–drug interactions, nor it is common practice to test the effect of all known inhibitors on the transport of a target drug. Extrapolation of inhibition data obtained with classical transporter inhibitors and translation to other inhibitors of the same transporter is challenging (e.g., due to different binding sites at the transporter), in particular in elderly patients who, as written above, receive multiple drugs.

P-glycoprotein 1 (P-gp, ABCB1), multidrug resistance-associated protein 2 (MRP2, ABCC2), and breast cancer resistant protein (BCRP, ABCG2) transporters are present in the bowel, liver, kidney, and blood–brain barrier (BBB). These transporters play a role in effluxing substrates back into the intestinal lumen, facilitating hepatic and renal excretion (except for BCRP), and functioning inversely at the BBB to protect the brain from foreign substances entry and to redirect the latter into the systemic circulation. Inhibition of their function in the intestine, liver, or kidney can lead to increased systemic exposure of substrates (although the opposite effect is expected when inhibiting transport across the BBB).

In the liver, kidney, and BBB, essential uptake transporters, such as organic cation transporters (OCTs) 1/2/3, organic anion-transporting polypeptides (OATPs) 1B1/1B3/2B1, and multidrug and toxic compound extrusion proteins (MATEs) 1/2, all follow a common route for transporting their substrates from the blood stream into various tissues, or into urine/bile. Consequently, modulating the capacities of these transporters would determine increased or decreased systemic drug concentrations. However, a contrary effect can be observed with the uptake transporters expressed in the apical membrane of the intestine, such as OATPs and OCT1, as the transportation pathway occurs in the opposite direction. In certain cases, altering the function of uptake transporters can increase the risk of ADRs. For instance, individuals with two reduced function alleles of OCT1 (SLC22A1) treated with OCT1 inhibitors were more than four times likely to experience gastrointestinal side effects during metformin (an OCT1 substrate) therapy, attributable to the accumulation of metformin in the intestinal lumen. This finding is supported by a previous study as well [112,113].

At the level of renal uptake transporters, other drug–drug-transporter gene interactions have been reported, where carrying mutant alleles and co-administration of inhibitors were associated with increased plasma levels/toxicity or reduced clearance of metformin [114,115]. On the other hand, reducing transport may decrease specific side effects. For instance, individuals carrying the rs316019 (C > A) mutation in OCT2 (SLC22A2) were protected against nephrotoxicity and ototoxicity caused by cisplatin (OCT2 substrate). The variant resulted in reduced transport of cisplatin into the kidneys and the inner ear (cochlea) where OCT2 is also expressed [116,117].

In many cases, the effectiveness of a drug depends on its ability to access certain tissues. Statins, for example, enter the liver by OATP1B1 (SLCO1B1), which is crucial for their lipid-lowering effects. Reducing this uptake pathway diminishes statin efficacy, leading to elevated plasma concentrations, and results in myopathy and, in rare occasions, rhabdomyolysis. The rs4149056 (T > C) variant (SLCO1B1*15) has been extensively studied and consistently associated with increased statin plasma exposure, muscle aches, dose reduction, and/or treatment-resistant phenotypes [118,119,120,121].

## 6. Strategies Supporting the Appropriateness of Drug Use and Prevention of ADRs in Elderly Patients

Refining drug therapy is an essential aspect of caring for elderly patients. The process of prescribing a medication includes specific information to be considered, such as drug indication, determining a dose, monitoring for effectiveness and toxicity, educating the patient about expected side effects, and indications for seeking consultation. Therefore, ADRs are the serious consequences of inappropriate drug prescribing [122].

As highlighted above, polypharmacy is a major risk factor for ADRs’ onset. Therefore, one of the most important interventions to reduce the threat of iatrogenic disease is to decrease the burden of medicines.

Deprescribing refers to the supervised process of discontinuing inappropriate medications or reducing their dosage enhancing patient outcomes [30,123,124]. *Scott* et al. propose a five-step protocol to facilitate this procedure. These steps involve conducting a review of the patient’s medications to assess their appropriateness in relation to clinical condition, overall functioning, life expectancy, and health priorities. Based on this evaluation, each drug should be carefully examined, bearing in mind the danger of ADRs and the benefit/risk ratio. Once the drug to be discontinued is identified, it is crucial to monitor for potential withdrawal reactions or improvements in outcomes [125].

Prescribing and deprescribing processes require meticulous documentation of the patient’s health status. This involves identifying clinical geriatric conditions, conducting a comprehensive review of medications (including herbal remedies and over-the-counter drugs), carefully analysing any previous ADRs, and establishing clear health priorities and treatment objectives. In older individuals with polypharmacotherapy, the introduction of new medicines should involve gradual titration to minimize the risk of adverse events, and any new symptom should be evaluated as potential ADRs [14]. This appears critical to prevent the occurrence of a prescribing cascade, which transpires when an additional medicine is prescribed to counteract an ADR that is mistakenly interpreted as a new medical condition. A classic example of this sequence is the prescription of anti-Parkinson drugs to manage motor symptoms associated with prolonged antipsychotic therapy [126].

In this scenario, adherence to therapy plays a key role. After determining the most suitable therapeutic approach, physicians should devote sufficient efforts and time to inform and engage patients, as well as their caregivers (e.g., family members or non-healthcare professionals responsible for the well-being of an elderly or dependent individual), and other healthcare professionals involved in their care. Effective physician–patient interaction is essential for enhancing the patient’s understanding of medical recommendations and promoting acceptance of the prescribed treatments [127].

Several studies have demonstrated that open communication between physicians and patients regarding diagnosis and treatment, thus incorporating shared decision making, improves adherence to medical advice and achieves positive short- and medium-term clinical outcomes [128,129,130]. In addition to ensuring that patients and caregivers are aware about necessity, role, and potential adverse effects of the prescription, acceptance may be influenced by various factors following treatment initiation. These determinants include the therapeutic benefits in terms of disease control and quality of life, the tolerability of medicines, and the convenience of drug administration according to formulation and dosage [131,132].

A facilitator of medication adherence is the establishment of interpersonal trust between physician and patient, essential in the patient–physician relationship, particularly among older patients. Research by *Thom* et al. has shown that low trust in physicians is associated with poorer adherence to medical recommendations, reduced satisfaction with care, and limited improvement in symptoms. Furthermore, when patients relay on their medical doctors, they are more likely to disclose their health-related behaviours, even those they may consider embarrassing or sensitive [133,134,135].

### 6.1. Tools to Identify Inappropriate Prescriptions

An appropriate prescription entails selecting the suitable medication treatment aligned with the patient’s specific requirements, ensuring an accurate dosage and duration. Several well-established tools are available to assess prescription appropriateness in older populations [50]. A recent systematic review gathered various published tools aimed at guiding clinicians in optimizing drug treatments for older individuals. Prominent examples include criteria such as the American Geriatrics Society (AGS) Beers criteria [136] and the Screening Tool of Older Persons’ Prescriptions (STOPP)/the Screening Tool to Alert to Right Treatment (START) criteria [137]. These widely recognized guidelines are developed through expert consensus processes, such as the Delphi method, and are periodically updated based on new evidence. Section A of the STOPP criteria defines general indications for the use of a medicine that may be inappropriate in patients over 65 years of age. Therefore, no drug may be prescribed without a clinical indication, beyond the recommended duration if this is well defined, and more than one drug from the same pharmacological class.

The other sections deal with individual apparatus or systems. For instance, section C concerns anticoagulants; with regard to clopidogrel, its combination with aspirin for long-term secondary prevention of stroke is not recommended unless the patient has undergone stenting in the previous 12 months, has a concomitant coronary syndrome or symptomatic carotid stenosis. Conversely, the START criteria refer to the potential omission of the prescription of certain drug therapies for clinically invalid reasons, assuming that the prescribing physician observes the specific contraindications. On this basis, for instance, the use of antiplatelet drugs is encouraged in the presence of a history of cerebrovascular, coronary, and peripheral vascular disease. Another example is the use of high-potency opioids in the treatment of moderate to severe non-arthritic pain when paracetamol, NSAIDs or milder opioids are inappropriate or ineffective [138]. Beers criteria are organised into five categories to identify PIMs in older people. A class of drugs that is often inappropriately taken is proton pump inhibitors; expert panel advice is to avoid prolonging use beyond 8 weeks except in high-risk patients, such as those on chronic anti-inflammatory treatment or with documented gastro-oesophageal disease. There would be a risk of *C. difficile* infection, pneumonia, gastrointestinal neoplasia, bone demineralisation and fractures [139].

On the other hand, the medication appropriateness index (MAI) utilizes a set of structured questions focused on factors like approved indications, evidence-based dosages, and the absence of duplications, without specifying particular medicaments. It is commonly employed during the medication review process [140].

Additionally, the Fit fOR The Aged (FORTA) List [141] categorizes medicinal products used for chronic diseases in older adults into four classes, based upon data regarding efficacy, safety, and suitability for the age group. Drugs included in these four categories can be graded as A (Absolutely) for indispensable, B (Beneficial) for clearly beneficial, C (Cautious) for controversial, and D (Do not) for completely unnecessary prescriptions. According to these classifications, FORTA-labelled medicines lists have been approved in seven European countries and the USA, reflecting the availability and use of medicines in each country [142,143,144].

Although many of these lists draw from similar evidence to classify drugs to be avoided in older adults, differences exist, and the prevalence of potentially inappropriate medications (PIMs) can vary significantly depending on the specific tool used. A recent study comparing the European Union Eu(7)-PIM list, Beers criteria, and STOPP criteria revealed poor agreement among these tools in identifying inpatients exposed to PIMs. Furthermore, the applicability of these tools in different settings and countries has only been extensively studied for a few criteria, such as Beers and STOPP/START.

Several country-specific criteria have been proposed in recent years to enhance their relevance to specific healthcare systems, particularly considering the absence of certain products in country-specific markets [145].

### 6.2. Medication Review and Medication Reconciliation

Regular assessment of drug regimens is essential. *The National Service Framework for Older People* emphasizes the importance of conducting treatment scheme evaluations for patients aged 75 years and older at least once annually. The purpose of a medication review, which involves a systematic and thorough examination of the individual therapeutic plan, is to optimize the effectiveness of medications and reduce the risk of adverse reactions. Furthermore, promoting effective communication among various healthcare providers is recommended to enhance a patient’s compliance and adherence [146,147].

The medication review process can be divided into different steps; trained nurses and clinical pharmacists may play active roles with clearly defined responsibilities. During hospital discharge or visits subsequent to secondary or primary care settings, both nurses and clinical pharmacists can assist physicians in ensuring that patients and caregivers understand the reasons for medication use, dosing instructions, duration of treatment, and other relevant information, thereby promoting adherence. Additionally, these professionals can be involved in monitoring medication adherence and evaluating specific outcomes related to the therapy benefit/risk profile, even without direct physician involvement, if appropriate local services are available [23].

Medication reconciliation, as defined by the *Institute for Healthcare Improvement*, provides the drafting of the most accurate list of a patient’s medications, including drug names, posology, frequencies, and routes of administration. This list is then compared against the physician’s orders during admission, transfer, and/or discharge within the hospital, with the aim of providing the patient with the correct medications at all transition points [148,149]. Implementing accurate and reliable medication reconciliation processes at all care transitions is compel for preventing ADRs and ensuring safety [150].

When a patient is admitted to a new healthcare facility, it is essential for clinicians to review the patient’s medication list along with new orders and care plans to reconcile any discrepancy. Accurate reconciliation requires information on drug names, dosages, frequencies, and administration route. The standardization of this process is important to ensure comprehensive reconciliation and reduce medication errors that can harm patients [151].

Medication reconciliation is also crucial during the discharge process from the hospital. Changes may have been made to medications during the hospital stay, including additions, modifications, holds, or discontinuations, due to factors such as newly diagnosed medical conditions, drug interactions, or compliance with the hospital’s formulary. Proper reconciliation of discharge medications is necessary to address these changes appropriately [152]. Although the medication review may potentially optimise pharmacotherapeutic regimen, to date no strong evidence for improvement in clinical outcomes emerged from literature. More encouraging data come from a systematic review which showed a consistent reduction in prescription discrepancies with medication reconciliation approach with a decrease in present and potential adverse drug reactions [153].

### 6.3. Drug Label Annotation Based on Pharmacogenetics

PharmGKB is a pharmacogenomics knowledge resource which supplies clinical information, including clinical guidelines and drug labels, potentially clinically actionable gene–drug associations, and genotype-phenotype relationships. It annotates drug labels containing pharmacogenetic information approved by the US Food and Drug Administration (FDA), European Medicines Agency (EMA), Swiss Agency of Therapeutic Products (Swissmedic), Pharmaceuticals and Medical Devices Agency, Japan (PMDA) and Health Canada (Santé Canada) (HCSC) [77].

The annotations are divided into four levels:

1. “Required genetic testing” refers to situations where labels indicate or imply that gene, protein, or chromosomal testing, including genetic testing, functional protein assays, or cytogenetic studies, should be carried out before initiating the treatment. Of note, testing may be necessary only for a specific subset of patients.
2. “Recommended genetic testing” concerning conditions in which labels indicate or imply that gene, protein, or chromosomal testing, including genetic testing, functional protein assays, or cytogenetic studies, is recommended prior the drug use. It is important to note that the recommendation may be applied only to a specific subset of patients.
3. “Actionable genetic testing” refers to labels that provide information about the influence of gene/protein/chromosomal variants or phenotypes on changes in drug efficacy, dosage, metabolism, or toxicity. These labels may also include specific contraindications of the drug for a subset of patients based on particular variants/genotypes/phenotypes.
4. “Informative genetic testing” is assigned to labels which yield information stating that specific gene/protein/chromosomal variants or metabolizer phenotypes have no impact on a drug’s efficacy, dosage, metabolism, or toxicity. Alternatively, these labels may indicate that although variants or phenotypes do affect a drug’s efficacy, dosage, metabolism, or toxicity, the effect is not clinically significant. This level is also assigned to all other labels that have been listed in the *FDA Table* but do not currently meet the criteria for all other PharmGKB annotations mentioned above.

Drug labels varies among Regulatory Agencies; indeed, from FDA it is possible to identify 137 drugs for which testing is required, 7 recommended, 146 actionable, and 140 informative. EMA indicates 87 required testing, 5 recommended, 45 actionable, and 71 informative. Swissmedic states 9 required testing, 5 recommended, 92 actionable, and 24 informative. For HCSC 69 testing are required, 6 recommended, 67 actionable, and 40 informative. PMDA identifies 14 required testing, 3 actionable, and 8 informative (see Table 2) [77].

Although most of the genetic tests reported by the Summary of Product Characteristic (SmPC) concern somatic mutations of genes encoding for molecular targets of antineoplastics, PharmGKB also reports genetic tests that may be performed to predict therapeutic response to other medication categories (i.e., codeine, antipsychotics). Moreover, the demographic increase of elderly population and the longer life expectancy means that cases and deaths from cancer will gradually increase worldwide among older adults. In addition, neoplasms in this category of patients have unique characteristics, particularly treatment outcomes, which may be affected by interaction mechanisms described above.

### 6.4. Digital Tools Supporting Appropriate Prescription

Nowadays, prescribing practices often rely on electronic tools that enable physician to simultaneously incorporate each prescription into the patient’s electronic health record and provide a receipt of it. This practice has led to the development of specific computerized prescription support systems, particularly beneficial for patients with comorbidities and polypharmacy, to aid in medication review and therapeutic decision-making. These systems fall under the broader category of digital health interventions (DHIs), which encompasses various technologies that facilitate meeting the health needs of single individuals and entire populations.

DHIs include e-Health (e.g., informative websites, educational videogames, telehealth webinars, digital therapeutics) and m-Health (e.g., mobile microsensors, voice marker analysis apps).

Physicians have access to numerous online resources, ranging from authoritative websites like deprescribing.org, which provide recommendations, videos, and lists of useful apps for specific therapeutic areas and user profiles, to software applications designed for computers or smartphones. These resources can be utilized for individual cases during patient visits or integrated with electronic chart databases to receive automatic warnings regarding potential inappropriate prescriptions or to periodically process prescription lists for each patient. Examples of such specific websites include medstopper.com [154], drugs.com [91], and intercheckweb.marionegri.it [155,156,157,158].

Moreover, computer software programs have been developed to assist physicians in prescribing and reduce ADRs while improving patient outcomes. Two of them, the Clinical Decision Support System (CDSS) and the Computerized Prescription Support System (CPSS), employing a variety of algorithms to identify potentially inappropriate prescriptions, assess the risk of iatrogenic illness, determine appropriate drug dosages, identify drug interactions, and highlight contraindicated treatments. Another one, the Computerized Provider Order Entry (CPOE), allows for healthcare providers to directly input orders into a computer, helping to detect and prevent potential errors [159,160].

However, the evidence supporting the impact of CPOE and CDSS on patient outcomes is limited, meaning that there are challenges in addressing and managing ADRs [161].

A randomized clinical trial demonstrated the effectiveness of CDSS in reducing undesired drug–drug combinations, although it revealed that this led to treatment delays in cases requiring immediate pharmacological therapy, resulting in the early termination of the study [162].

**Figure 2:**
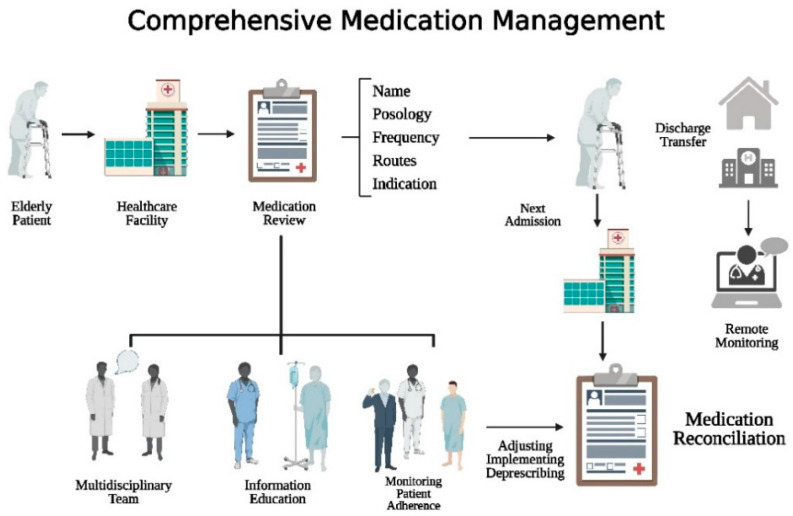
Management for prescription appropriateness in elderly patients. Created with BioRender.com; accessed on 10 September 2023.

A graphic representation of the elderly patient’s suggested pathway from access to the healthcare facility to therapy adjustment is shown in Figure 2.

## 7. Conclusions

The complexity of medical conditions observed in older patients emphasizes the compelling necessity of adopting a comprehensive approach to their care. The latter is particularly crucial when dealing with high-risk populations, such as residents in long-term care facilities or frail, multimorbid older adults who are hospitalized. Despite the development of various tools aimed at mitigating the risk of adverse drug reactions, prevention remains a significant challenge. The reliance on disease-specific guidelines for managing individual conditions is still prevalent, often placing older individuals with multiple health issues at a disadvantage and increasing chances of ADRs. To alleviate the burden of ADRs, interventions focused on pharmaceutical principles, such as medication review and reconciliation, may be integrated into a broader assessment of patients’ characteristics, needs, and health priorities.

In this line, digital health interventions could offer valuable solutions to assist healthcare professionals in identifying inappropriate prescriptions and promoting patient adherence to treatment plan. While the effectiveness of each specific strategy is still not thoroughly supported by evidence, particularly in terms of primary clinical outcomes and health-economic sustainability, a widespread recognition among physicians, patients, and policymakers arises regarding the necessity for integrated approaches.

Furthermore, a key role in this field may be played by pharmacogenetics/genomics since it could represent a cornerstone in moving patient care from the conventional approach to precision medicine.

## Figures and Tables

**Table 1:** Classification methods for adverse drug reactions.

Thomson and Rawlins	Type A (Augmented)	Type B (Bizarre)	Type C (Continuing)	Type D (Delayed)	Type E (End-of-Use)	Type F (Failure)
	Response to drugs administrated at therapeutic doses being the result of an abnormal response of an otherwise normal pharmacological effect.	Unrelated to the pharmacodynamics or the dosage of the drug and are often fatal. These are less common, and so may only be discovered for the first time after a drug has already been made available for general use.	Related to the cumulative dose of a long-term pharmacological treatment.	Consequence to the timing of a treatment and become apparent sometime after the use of a medicine.	Associated to the withdrawal of a given medicine.	Occurring when a therapy appears futile.
**Dose, Time and Susceptibility (DoTS)**	**Relation to Dose (Do)**		**Time Course (T)**		**Susceptibility Factors (S)**	
	▪ Toxic reactions. ▪ Collateral reactions. ▪ Hyper-susceptibility reactions.		▪ Time-independent reactions. ▪ Time-dependent reactions.		▪ Genetic ▪ Age ▪ Sex ▪ Physiological variation ▪ Exogenous factors ▪ Diseases	
**EIDOS**	**Extrinsic chemical species (E)**	**Intrinsic chemical species (I)**	**Distribution (D)**		**Outcome (O)**	**Sequelae (S)**
	This can be the parent compound, an excipient, a contaminant or adulterant, a degradation product or a derivative of any of these.	This is usually the endogenous molecule with which the extrinsic species interacts; this can be a nucleic acid, an enzyme, a receptor, an ion channel or transporter or some other protein.	A drug will not produce an adverse effect if it is not distributed to the same site as the target species that mediates the adverse effect. Thus, the pharmacokinetics of the extrinsic species can affect the occurrence of adverse effects.		Interactions between extrinsic and intrinsic species in the production of an adverse effect can result in physiological or pathological changes.	The sequela of the changes induced by a drug describes the clinically recognizable adverse drug reaction, of which there may be more than one.

**Table 2:** PharmGKB summary of drug label annotations among Regulatory Agencies.

	Required Genetic Testing	Recommended Genetic Testing	Actionable Genetic Testing	Informative Genetic Testing
**FDA**	137	7	146	140
**EMA**	85	5	45	71
**Swissmedic**	9	5	92	24
**HCSC**	69	6	67	40
**PMDA**	14	/	29	8

## Data Availability

Data sharing is not applicable.
